# Antimicrobial and anti-biofilm activities of *Lactobacillus kefiranofaciens* DD2 against oral pathogens

**DOI:** 10.1080/20002297.2018.1472985

**Published:** 2018-05-28

**Authors:** Dana Jeong, Dong-Hyeon Kim, Kwang-Young Song, Kun-Ho Seo

**Affiliations:** Center for One Health, College of Veterinary Medicine, Konkuk University, Hwayang-dong, Gwangjin-gu, Seoul05029, Korea

**Keywords:** Kefir, *Lactobacillus kefiranofaciens*, oral probiotics, oral biofilm, *Streptococcus mutans*, *Streptococcus sobrinus*, dental caries, artificial oral model system

## Abstract

**Background**: *Streptococcus mutans* and *Streptococcus sobrinus* are major causative bacterial pathogens of dental caries.

**Objective**: We investigated the applicability of three *Lactobacillus* strains (*L. kefiranofaciens* DD2, DD5, and DD6) isolated from kefir and three commercial *Lactobacillus* strains (*L. plantarum* ATCC 10,012, *L. johnsonii* JCM 1022, and *L. rhamnosus* ATCC 7469) as potential oral probiotics with respect to their survivability in an experimental oral environment, antimicrobial activity, and anti-biofilm formation activity against *S. mutans* and *S. sobrinus*.

**Results**: Strains DD2, ATCC 10012, ATCC 7469, and JCM 1022 had the best oral survivability, including aerotolerance and enzymatic resistance, and inhibited the growth and biofilm formation of *S. mutans* and *S. sobrinus*. In particular, DD2 suppressed all three classes of biofilm formation-associated genes: those associated with carbohydrate metabolism and those encoding regulatory biofilm and adhesion proteins.

**Conclusions**: These results indicate that the novel kefir isolate *L. kefiranofaciens* DD2 effectively and directly inhibits *S. mutans* and *S. sobrinus*.

## Introduction

Probiotics are live microorganisms or bacterial cultures that can have beneficial effects for the host when ingested [1], including growth inhibition of pathogenic bacteria, modulation of intestinal microbiota, and suppression of low-grade systemic inflammation [2]. These effects are thought to be exerted via enhancement of intestinal functions or inhibition of metabolic diseases caused by intestinal dysbiosis [3,4]. Despite the fact that the oral cavity is the first point of contact between probiotics and the host system [5], few studies have directly investigated the effects of probiotics on the oral cavity.

Members of the *Streptococcus* genus, especially *S. mutans* and *S. sobrinus* [6,7], are the main causal pathogens of early dental caries owing to their ability to produce insoluble glucan and fructan and to attach to the tooth surface [8]. Streptococci are also known to aggregate to form oral biofilms; for instance, *S. mutans* rapidly increases biofilm thickness by producing surface-associated and biofilm regulatory proteins [7,9,10]. As such, reducing the size of cariogenic bacterial populations and suppressing biofilm formation are essential strategies for the prevention of oral diseases [11].

Antibiotics such as erythromycin, metal salt, and fluoride have conventionally been used to target cariogenic bacteria in the oral cavity [12]. However, these drugs are associated with negative side effects such as tooth discoloration and irritation [12]. As an alternative, some studies have investigated the anticariogenic role of probiotic microorganisms, including lactic acid bacteria (LAB) [13–15].

Kefir, one of the most popular sources of probiotics, is made by fermenting milk with multiple microorganisms [16]. Indeed, kefir contains over 50 species of LAB, yeasts, and acetic acid bacteria as well as their metabolites such as lactic acid, exopolysaccharides, and peptides that confer various beneficial health effects [2,16–18]. *Lactobacillus kefiranofaciens* is a typical probiotic microorganism found in kefir [4], and we previously demonstrated its antimicrobial effects against pathogenic bacteria *in vitro* and its ability to improve the balance of intestinal microbiota by reducing the proportion of opportunistic pathogens [4,18]. However, no study has investigated the inhibitory effect of kefir isolates against oral pathogens and the associated mechanisms.

Accordingly, in the present study, we investigated the applicability of *L. kefiranofaciens* isolated from kefir as a potential oral probiotic and compared its inhibitory effects against pathogenic oral bacteria to those of commercial LAB strains. We screened candidate oral probiotics based on their recovery rates under aerobic and anaerobic conditions and their survivability in an artificial oral environment, and determined their antimicrobial and anti-biofilm-forming activities against two major cariogenic pathogens (*S. mutans* and *S. sobrinus*). We also examined the mechanism underlying the inhibitory effects of the probiotics on these oral pathogens by evaluating changes in the expression of genes associated with biofilm formation.

## Materials and methods

### Experimental design

**Figure 1.**
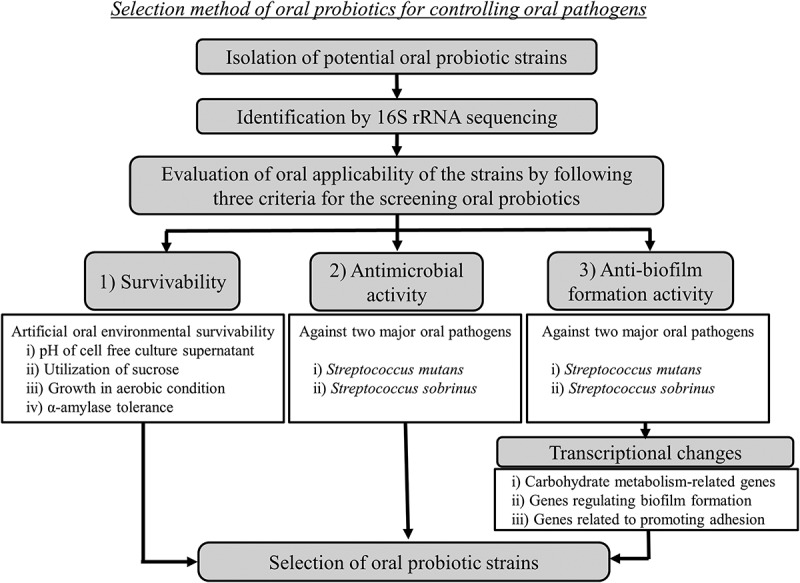
Flow diagram of the experimental procedures and design.

A schematic diagram of the experimental procedures used for selecting oral probiotics among isolated LABs for the assessment of mechanisms is shown in Figure 1.

### Bacterial strains

A total of three kefir isolates were used in this study, which were obtained from our private collection and are stored at –70°C. To recover the cryopreserved strains, a loop of thawed cryoprotactant solution was streaked onto de Man, Rogosa, and Sharpe (MRS) agar (Difco, Detroit, MI, USA), incubated anaerobically at 37°C for 72–96 h, and subcultured twice. In addition, a total of three commercial strains were obtained from the Korean Collection for Type Cultures (Daejeon, Korea): *Lactobacillus plantarum* ATCC 10012, *Lactobacillus johnsonii* JCM 1022, and *Lactobacillus rhamnosus* ATCC 7469. Each strain was subcultured twice before use in the experiment. All strains used are listed in Table 1.

**Table 1.** Screening of six lactic acid bacterial strains as candidate oral probiotics.*

			pH of cultured MRS broth			Growth under aerobic conditions (37°C, 48 h)		
Strain		Species (16S rRNA gene sequence identity %)	24 h	48 h	Sucrose utilization	Colony size^1^	Colony count^2^	α-Amylase tolerance (MRS, pH 6.8, 1,000 IU/ml of α-amylase, 37°C, 4 h)^3^
Kefir isolates	DD2	*Lactobacillus kefiranofaciens* (99.9)	6.06 ± 0.01	4.15 ± 0.02	−	NS	+++	+++
	DD5	*Lactobacillus kefiranofaciens* (99.9)	6.14 ± 0.02	4.39 ± 0.04	−	*D*	++	++
	DD6	*Lactobacillus kefiranofaciens* (99.9)	6.11 ± 0.01	4.37 ± 0.02	−	*D*	++	++
Commercial probiotic strains	ATCC 10012	*Lactobacillus plantarum* (99.9)	4.15 ± 0.03	3.83 ± 0.05	+	NS	+++	++
	JCM 1022	*Lactobacillus johnsonii* (99.9)	4.06 ± 0.01	3.87 ± 0.02	+	*D*	+++	+++
	ATCC 7469	*Lactobacillus rhamnosus* (99.9)	3.97 ± 0.02	3.86 ± 0.02	-	NS	+++	++

*Strains were identified based on 16S rRNA gene sequence homology and were characterized according to the pH of MRS broth cultures, sucrose utilization, and growth under aerobic conditions. α-Amylase tolerance was assessed in modified MRS broth. Three independent experiments were performed. ^1^NS, not significant; *D*, colony diameter <75% of that detected in anaerobic cultures. ^2^+++, >90% survival; ++, >50% survival; +, <50% survival; −, no survival vs. colony count under anaerobic conditions. ^3^+++, >90% survival; ++, >50% survival; +, <50% survival; −, no survival vs. initial count.

### Sequencing and identification of LABs

Partial sequences of the 16S ribosomal RNA gene were amplified from the genomic DNA of the strains and sequenced according to the protocol described in our previous study [18]. Briefly, genomic DNA extraction from the isolates was performed using the NucliSENS easyMAG instrument (bioMérieux, Marcy l’Etoile, France) according to the manufacturer’s instructions. Following polymerase chain reaction (PCR) amplification of the 16S ribosomal RNA gene, the amplicon was sequenced in an Applied Biosystems 3730xl DNA Analyzer at Cosmo Genetech (Seoul, Korea). The identities of the isolates were determined on the basis of the highest BLAST score (http://www.ncbi.nlm.nih.gov.blast) against the GenBank DNA database (www.ncbi.nlm.nih.gov/Genbank/).

### Investigating the applicability of candidate strains as an oral probiotic

All kefir isolates and commercial LAB strains were assessed in terms of the following characteristics: pH of the culture, sucrose utilization, growth under aerobic conditions, and α-amylase tolerance. The pH of the culture supernatant was measured after incubation for 24 and 48 h using a Model 205 pH meter (Testo, Lenzkirch, Germany) equipped with temperature compensation to determine the extent of acid production. Prior to measurement, the strains were diluted in sterilized 0.9% saline at a turbidity of 0.6 McFarland, and 100 µl of the diluent was inoculated into 3 ml of MRS broth, which was incubated at 37°C for 48 h. Sucrose utilization was determined through automated biochemical analysis using the Vitek 2 system (bioMérieux) to evaluate the potential of the candidate strains to compete with oral pathogens for sucrose, which is the major carbon source of oral streptococci [13].

We also compared the growth characteristics of each strain under aerobic and anaerobic conditions to evaluate their survivability in an experimental oral environment [19]. The test strains were diluted in sterilized 0.9% saline and spread onto two MRS agar plates that were aerobically or anaerobically incubated at 37°C for 48 h. At the end of incubation, the number and diameter of the colonies on the two plates were compared. Survivability was considered to be reduced if colonies from the aerobically grown cultures had a diameter that was <75% compared to that of colonies from anaerobic cultures.

Finally, α-amylase tolerance was assessed as a measure of resistance to oral enzymatic stress. For this assay, the pH of the MRS broth was adjusted to 6.8 and the broth was supplemented with 1000 IU/ml α-amylase from human saliva (Sigma, St. Louis, MO, USA) to mimic the environment of the oral cavity [20]. The strains were cultured in 3 ml of modified MRS broth and incubated for 0 (initial sample) or 4 h at 37°C. Samples were serially diluted 10-fold in 0.05 M sodium phosphate buffer (pH 7.0), and the number of viable LAB growing on the MRS agar was counted. α-Amylase tolerance was then determined by comparing the final plate count after 4 h with the initial plate count at 0 h. The experiment was performed three times.

### Antimicrobial activity of the LAB culture supernatant against oral pathogens

To investigate the antimicrobial effect of the LAB supernatants, the antimicrobial activity against two major oral pathogens was investigated by growth curve analysis according to our previous study [2]. The three kefir isolates and three commercial strains, respectively, were cultured in MRS broth at 37°C for 72 h. After centrifugation at 3134 × *g* for 20 min at 4°C, the supernatant was filter sterilized with a 0.45-μm syringe filter (Millipore, Bedford, MA, USA). The pathogens *S. mutans* ATCC 25175 and *S. sobrinus* ATCC 33478 (American Type Culture Collection, Manassas, VA, USA) were cultured on nutrient agar (Oxoid, Basingstoke, Hampshire, UK) for 24 h at 37°C for two passages and then used for growth curve analysis. A 100-μl aliquot of the culture supernatant was added to 1 ml of nutrient broth, and the mixture was inoculated with a 50-μl suspension containing 10^5^–10^6^ colony-forming units (CFUs) of *S. mutans* and *S. sobrinus* each. After mixing, 200 µl of each sample was transferred to a 96-well plate, and growth curves were generated at 1-h intervals over 24 h by measuring the optical density at 595 nm using a Multiskan FC microplate reader (Thermo Fisher Scientific, Waltham, MA, USA) at 37°C. The procedure was repeated three times for each sample. Based on the antimicrobial activity, one kefir isolate (DD2) and three commercial strains (ATCC 10012, JCM 1022, and ATCC 7469) were selected for the subsequent anti-biofilm formation assay.

### Biofilm formation of the culture supernatant of selected LAB against two oral pathogens

The effect of the LAB supernatant on biofilm formation was evaluated as previously described [21], with slight modification. In brief, the culture supernatant of the kefir isolate and three commercial strains were prepared as described above. A 100-μl aliquot of the supernatant was added to 100 µl nutrient broth containing 10^5^–10^6^ CFU of *S. mutans* and *S. sobrinus* each, and the control sample was prepared by adding 100 µl MRS medium instead of the culture supernatant. A 200-µl volume of each sample was transferred to a 96-well polystyrene culture plate following incubation at 37°C for 24 h. To assess the extent of biofilm formation in each microplate, the culture medium was discarded and the microplate was washed twice with 200 μl of phosphate-buffered saline (PBS). Adherent biofilm cells were stained with 200 μl of 0.1% (w/v) crystal violet for 15 min and rinsed twice with PBS. After removing the bound dye from stained cells with 200 μl of 99% ethanol, the amount of biofilm was quantified by measuring the absorbance of the solution at 595 nm with a spectrophotometer. Three replicates were prepared for each sample.

### Gene expression in the supernatant of selected LAB cultured with oral pathogens

To investigate the underlying anti-biofilm formation mechanism, we evaluated the mRNA levels of *S. mutans* genes encoding virulence proteins related to carbohydrate metabolism (*ftf, gtfB*, and *gtfC*), biofilm formation (*brpA, comDE*, and *vicR*), and adhesion (*gbpB* and *spaP*) by reverse transcription real-time PCR as previously described [22]. Following growth curve analysis, total RNA was extracted from the incubated samples using NucliSENS easyMAG (bioMérieux) and RNase-free DNase I (Takara Bio, Otsu, Japan) according to the manufacturer instructions. The RNA concentration was determined with a Nanodrop ND-2000 spectrophotometer (Thermo Fisher Scientific). The cDNA was prepared by reverse transcription using the PrimeScript RT Reagent kit (Takara Bio) and amplified by real-time quantitative PCR using SYBR Premix Ex Taq (Takara Bio) on an ABI 7500 system (Applied Biosystems, Foster City, CA, USA) with the primer sets listed in Table 2. The real-time PCR conditions were as follows: 95°C for 30 s, followed by 40 cycles of 95°C for 5 s and 60°C for 34 s. For melting curve analysis, the temperature was decreased from 95°C to 65°C at a rate of 0.1°C/s with continuous acquisition of the fluorescence signal intensity. Data were analyzed using ABI 7500 v.2.3. software (Applied Biosystems) and differences in mRNA expression levels were calculated after normalizing to the 16S rRNA level. Results are expressed as the fold change relative to that of the control group, and fold changes ≤0.5 or ≥1.5 were considered significantly different.

**Table 2.** Group-specific primer sets used for quantitative reverse transcription real-time PCR.

Function	Target gene	Primers	Sequence (5ˊ–3ˊ)	Reference
Carbohydrate metabolism-promoting genes	*ftf*	For	AAATATGAAGGCGGCTACAACG	[6]
		Rev	CTTCACCAGTCTTAGCATCCTGAA	
	*gtfB*	For	AGCAATGCAGCCAATCTACAAAT	[8]
		Rev	ACGAACTTTGCCGTTATTGTCA	
	*gtfC*	For	GGTTTAACGTCAAAATTAGCTGTATT	[8]
		Rev	AGC CTCAACCAACCGCCACTGTT	
Regulatory protein-encoding genes	*brpA*	For	GGAGGAGCTGCATCAGGATTC	[6]
		Rev	AACTCCAGCACATCCAGCAAG	
	*comDE*	For	ACAATTCCTTGAGTTCCATCCAAG	[6]
		Rev	TGGTCTGCTGCCTGTTGC	
	*vicR*	For	TGACACGATTACAGCCTTTGATG	[6]
		Rev	CGTCTAGTTCTGGTAACATTAAGT CCAATA	
Adhesion-promoting genes	*gbpB*	For	ATGGCGGTTATGGACACGTT	[6]
		Rev	TTTGGCCACCTTGAACACCT	
	*spaP*	For	GACTTTGGTAATGGTTATGCATCAA	[6]
		Rev	TTTGTATCAGCCGGATCAAGTG	
Housekeeping gene	*16S rRNA*	For	CCTACGGGAGGCAGCAGTAG	[6]
		Rev	CAACAGAGCTTTACGATCCGAAA	

### Statistical analysis

Data are reported as the mean ± standard deviation (SD) and were analyzed with SPSS v.19.0 software (SPSS Inc., Chicago, IL, USA) and Prism 6.01 software (GraphPad Inc., San Diego, CA, USA). Bacterial counts (CFU) in each sample were converted to log_10_ CFU/g, and mean values were compared with the Student *t* test. Differences were considered significant at *P* < 0.05. Data were also evaluated by analysis of variance (with the Tukey post-hoc test), with *P* < 0.05 considered significant.

## Results

### Isolation of L. kefiranofaciens from kefir and screening of oral probiotic strains

Three LAB strains were isolated from kefir (Table 1), and the complete sequences of their 16S rRNA genes were compared to those in the GenBank DNA database. The sequences were 99.9% identical to those of *L. kefiranofaciens* (Table 1).

As shown in Table 1, the initial pH value of the cultures was 6.8, but decreased to 3.97–4.15 for commercial strains and to 6.06 or higher for the kefir isolates after 24 h of incubation. After 48 h, the culture pH of the commercial strains was significantly lower than that of the kefir isolates, which ranged from 4.15 to 4.39 (*P* < 0.05). All tested strains were negative for sucrose utilization except for ATCC 10012 and JCM 1022. In the aerotolerance test, there were no differences in colony diameter between growth under aerobic and anaerobic conditions for strains DD2, ATCC 10012, and ATCC 7469. Strains DD2 and JCM 1022 showed the highest amylase resistance, with survivability >90%.

### Antimicrobial activity of LAB culture supernatants against oral pathogens

**Figure 2.**
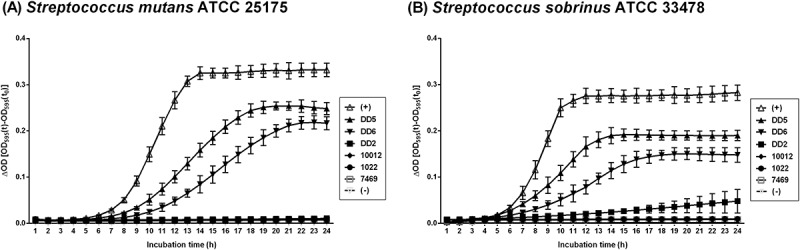
Growth of *Streptococcus mutans* ATCC 25175 (A) and *Streptococcus sobrinus* ATCC 33478 (B) in nutrient broth (NB) mixed with the spent culture supernatant from each candidate probiotic strain.

The antimicrobial effect of the culture supernatants of the selected strains against two major oral pathogens was evaluated by measuring the optical densities of the cultures at 595 nm (OD_595nm_). The unsupplemented group showed a steady increase in the OD_595nm_ value up to 0.43. *S. mutans* growth was completely inhibited by the culture supernatants of DD2, ATCC 10012, JCM 1022, and ATCC 7469, with partial suppression achieved by the DD5 and DD6 supernatants to <0.30 at OD_595nm_ (Figure 2A). Similar results were obtained for *S. sobrinus*: while the OD_595nm_ increased in the unsupplemented group to 0.29, growth was completely inhibited by addition of the culture supernatants of DD2, ATCC 10012, JCM 1022, and ATCC 7469 (Figure 2B). Furthermore, the maximum growth of *S. sobrinus* was inhibited to <0.2 at OD_595nm_ in the presence of the DD5 and DD6 supernatants.

### Inhibition of biofilm formation by the culture supernatants of the selected LAB

**Figure 3.**
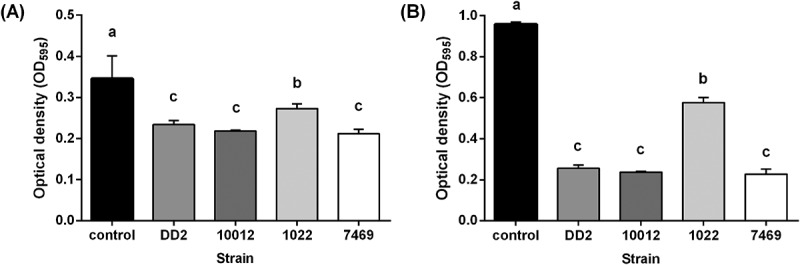
Inhibition of *Streptococcus mutans* ATCC 25175 biofilm formation by culture supernatants of kefir-derived and commercial probiotic strains. Biofilm formation was assayed on polystyrene microtiter plates after staining with crystal violet. Three independent experiments were performed. Error bars represent standard deviations.

The four strains that completely inhibited the growth of the two oral pathogens were further investigated for their effects on biofilm formation. In contrast to the control group, the supernatants of all four LAB significantly reduced biofilm formation by *S. mutans* and *S. sobrinus* (Figure 3A, B).

### Gene expression profiles of the culture supernatants of the selected LAB against oral pathogens

The fold changes in the *S. mutans* mRNA expression levels caused by the selected probiotic strains are shown in Table 3. The expression of *ftf*, which is associated with carbohydrate metabolism, was decreased by more than 0.29-fold in the DD2-supplemented groups. In addition, DD2 decreased the expression levels of genes encoding regulatory proteins: 0.41-fold for *brpA*, 0.21-fold for *comDE*, and 0.23-fold for *vicR*. Expression of the *vicR* gene was also downregulated by 0.13-fold in the ATCC 7469-supplemented groups. The expression levels of the adhesion-related genes *gbpB* and *spaP* were also reduced relative to those of the control group by the supernatants of strains DD2, JCM 1022, and ATCC 7469. Overall, these findings indicate that the DD2 strain was most effective in suppressing the expression of genes related to biofilm formation by oral pathogens, influencing all three categories of inhibition evaluated.

**Table 3.** Fold changes in the expression of eight genes associated with biofilm formation.

				Fold change relative to control^a,b^			
Category	Gene symbol	Gene description	Function	DD2	ATCC 10012	JCM 1022	ATCC 7469
Carbohydrate metabolism-promoting genes	*ftf*	Fructosyltransferase	Synthesis of fructan polymers from sucrose	0.29*	1.66	0.53	0.67
	*gtfB*	Glucosyltransferase B	Synthesis of adhesive extracellular glucans from sucrose	0.73	1.81	0.74	0.51
	*gtfC*	Glucosyltransferase C	Synthesis of adhesive extracellular glucans	0.64	1.60	0.81	0.90
Regulatory protein-encoding genes	*brpA*	Biofilm regulatory protein A	Response to environmental stress and biofilm development – putative surface-associated polypeptide	0.41*	1.87	1.65	1.39
	*comDE*	Competence-stimulating peptide	Regulation of genetic transformation and biofilm formation	0.21*	1.78	1.54	0.57
	*vicR*	Histidine kinase two-component regulatory system	Modulation of adherence and biofilm formation, and regulation of the expression of virulence-associated genes	0.23*	1.03	0.78	0.13*
Adhesion-promoting genes	*gbpB*	Glucan-binding protein	Adhesion to glucans and promotion of plaque formation	0.30*	0.67	0.44*	0.29*
	*spaP*	Cell wall-associated adhesin P1 or multi-functional adhesin	Sucrose-independent adhesion	0.14*	0.52	0.36*	0.28*

DD2, supplemented with DD2 culture supernatant; 10,012, supplemented with ATCC 10,012 culture supernatant; 3141, supplemented with JCM 1022 culture supernatant; 7469, supplemented with ATCC 7469 culture supernatant.

^a^Control, *Streptococcus mutans* cultured in unsupplemented NB for 24 h.

^b^Fold change ≥1.5 or ≤0.5 was considered statistically significant, indicated by *.

## Discussion

In the present study, we isolated the LAB from kefir and compared their applicability as oral probiotics relative to the antimicrobial and survival properties of commercial strains. Collectively, our results suggest that *L. kefiranofaciens* DD2 potentially can be developed as an effective probiotic product for combating dental caries.

Although many studies have explored the use of LAB for the benefit of oral health, there are currently no comprehensive or standardized *in vitro* protocols for screening novel oral probiotics [5,13,23]. Probiotic candidates have been routinely screened based on their ability to survive in an artificial host barrier system such as with stimulation of gastric acid or enzymatic stresses [4]. In this study, we assessed novel aspects in probiotic screening using an experimental oral environment, including resistance against ambivalent atmospheric conditions in a survival assay and enzymatic action towards utilization of sucrose and acid production, which are linked to the development of dental caries.

Given that the presence of oxygen is a major factor in bacterial survival, we investigated growth characteristics under both aerobic and anaerobic conditions. In general, several heterofermentative lactobacilli grow better in the presence of oxygen [24]. However, adaptation to aerobic conditions is strain specific [25], which was also demonstrated in the present study given the growth inhibition of DD5 and DD6 but not DD2.

The survivability of the candidate strains was evaluated in a modified MRS medium containing α-amylase, which is a major oral digestive enzyme involved in the breakdown of polysaccharides that can reduce the survival of probiotic strains in the oral cavity by attacking bacterial polysaccharide components [26]. The commercial strains and those isolated from kefir were all highly resistant to α-amylase activity, which may be due to the action of capsular materials or protective polymeric substances surrounding the cell [18].

In addition, both the kefir isolates and the commercial strains showed strong antimicrobial activity against typical cariogenic bacteria. The antimicrobial activity of kefir or its constituent microorganisms against various foodborne pathogens and spoilage bacteria has been reported previously [2,27]; however, the actual antimicrobial properties against cariogenic bacteria have not previously been demonstrated. LAB produce various antimicrobial compounds such as organic acids, hydrogen peroxide, diacetyl, and bactericidal polysaccharides or peptides [28]. The antimicrobial activity of LAB can be attributed to their ability to produce organic acid – which lowers the pH of the culture supernatant – as well as to their antibacterial substances [29]. Indeed, strains that lowered the supernatant pH by a larger margin showed greater inhibition of both *S. mutans* and *S. sobrinus*. Notably, the culture supernatant of *L. kefiranofaciens* DD2 exhibited antimicrobial activity despite a relatively high pH as compared to the commercial strains, implying that other substances contribute to the inhibitory effects [18,29]. Indeed, in our previous study, we found that *L. kefiranofaciens* produces an antimicrobial exopolysaccharide [18], which could be linked to the antimicrobial action; however, additional studies are needed to investigate the mechanism of action.

Among the four strains showing antimicrobial activity, *L. kefiranofaciens* DD2 exhibited the strongest anti-biofilm formation activity. Various clinical studies have shown that *L. rhamnosus* and *L. paracasei* consumption could reduce *S. mutans* biofilm formation in the oral cavity [15,30]. Despite the extensive research effort devoted to determining the effects of LAB supplementation on cariogenic bacteria, there are still large knowledge gaps related to understanding the mechanism of the bacterial behavior in response to supplementation. A recent study demonstrated that *Lactobacillus acidophilus* downregulates the *gtf* gene of *S. mutans* [8]; however, the molecular mechanisms have only been assessed thus far by focusing on one or two genes of interest. This is the first study to simultaneously investigate more than two different genes associated with biofilm formation as a potential mechanism of pathogenic bacteria inhibition.

Recent studies have emphasized the importance of biofilm formation in the development of dental caries [31–33]. It is well accepted that dental caries is a representative biofilm-dependent oral disease, and complex interactions among specific oral microorganisms, host factors, and diet that accelerate the establishment of cariogenic biofilms on tooth surfaces are deeply involved in the underlying etiology [31,32]. In these processes, *S. mutans* is not always the most abundant taxon, but is a key matrix producer and modulator of cariogenic biofilms in the oral cavity [33]. Thus, the current platforms for the selection of anticariogenic oral probiotics essentially include the anti-biofilm activity of the candidates, which could be investigated more in depth by coupling with various molecular analyses [32].

**Figure 4.**
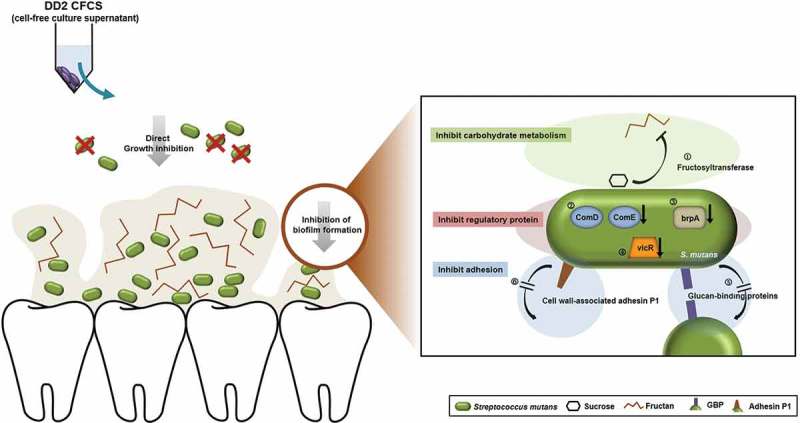
Schematic representation of the dual-inhibition mechanism of oral streptococci by *Lactobacillus kefiranofaciens* DD2. ComD, competence-stimulating peptide D; ComE, competence-stimulating peptide E; brpA, biofilm regulatory protein A; vicR, histidine kinase two-component regulatory system; GBP, glucan-binding proteins.

Biofilm formation involves many factors associated with carbohydrate metabolism, adhesion, and regulation [34,35]. The *ftf* gene encodes fructosyltransferase, which synthesizes fructan polymers that act as binding sites for *S. mutans* and thereby promote biofilm formation [36] (Figure 4). Thus, our results demonstrated that supplementation with the DD2 culture supernatant could influence fructan production in *S. mutans*. Furthermore, the downregulation of *ftf* was accompanied by decreases in the expression levels of *comDE* and *brpA* (Figure 4), which are also involved in the biofilm formation and play a critical role in the regulation of stress responses [9,37]. Interestingly, the expression level of *vicR* (Figure 4) – which encodes proteins that regulate the expression of virulence factors involved in polysaccharide synthesis and those that integrate external signals in a regulatory network composed of *ftf, gtfB*, and *gtfC* [37] – was significantly decreased. Thus, DD2 inhibits *S. mutans* biofilm formation and stability by suppressing the expression of key regulatory factors.

The production of carbohydrates as well as their accumulation on the tooth surface is important for biofilm formation. Surface proteins that cause oral bacterial aggregation include glucan-binding proteins and adhesion P1 (Figure 4), which have high affinity for microorganisms and the tooth surface and thereby promote plaque formation [7,35]. We found that the DD2 culture supernatant reduced the expression levels of both genes, suggesting that DD2 also inhibits the aggregation step of biofilm formation.

In conclusion, *L. kefiranofaciens* DD2 showed three oral probiotic attributes in an artificial oral model system: (i) excellent oral survivability, (ii) growth inhibition against oral streptococci, and (iii) anti-biofilm formation capacity against oral streptococci via inhibition of associated genes. These findings indicate that *L. kefiranofaciens* DD2 potentially can be developed as a novel oral probiotic agent.

## Supplementary Material

Supplemental Material
