# Supplementary material for: Antimicrobial and anti-biofilm activities of Lactobacillus kefiranofaciens DD2 against oral pathogens
Source: J Oral Microbiol. 2018 May 28;10(1):1472985. doi: 10.1080/20002297.2018.1472985 (PMC5974711; doi:10.1080/20002297.2018.1472985)
Supplement: Supplemental Material [file ZJOM_A_1472985_SM6939.docx]

Supplementary data 1. The amplification plot and dissociation curve of the real-time PCR assay targeting 1) *ftf*, 2) *gtfB*, 3) *gtfC*, 4) *brpA*, 5) **comDE**, 6) *vicR*, 7) **gbpB**, 8) **spaP**, and 9) *16S rRNA* gene.

1) *ftf* gene

| Amplification plot | Dissociation curve |
| --- | --- |
| 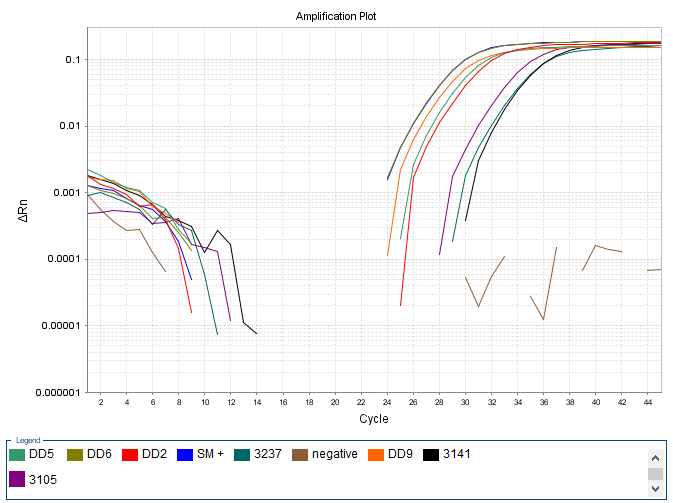 | 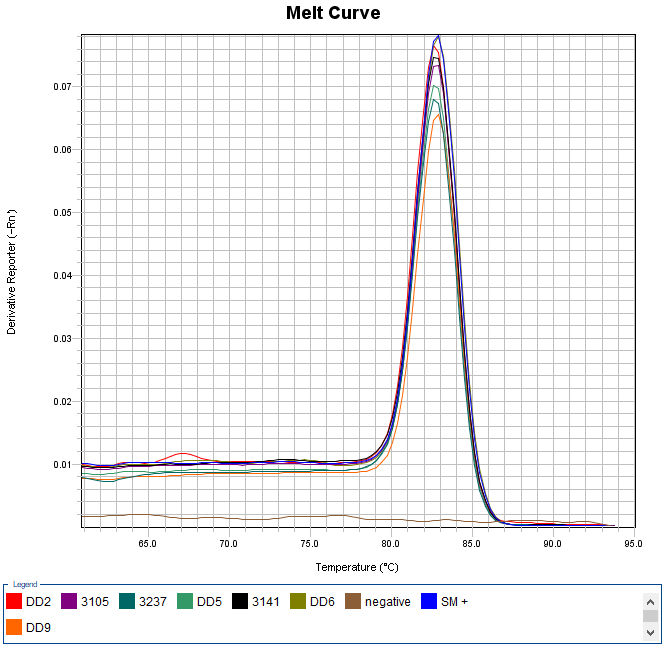 |

2) *gtfB* gene

| Amplification plot | Dissociation curve |
| --- | --- |
| 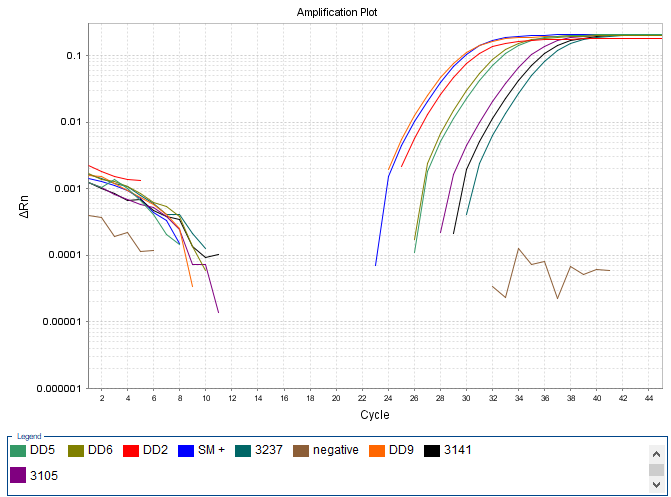 | 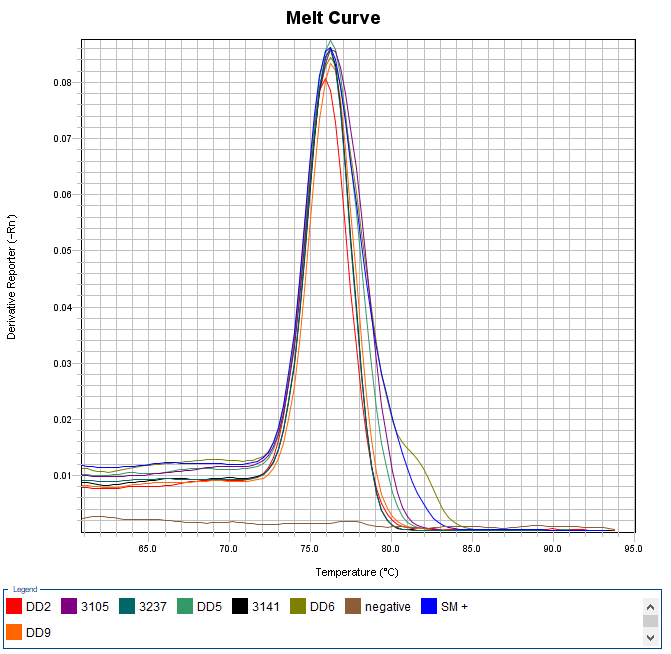 |

3) *gtfC* gene

| Amplification plot | Dissociation curve |
| --- | --- |
| 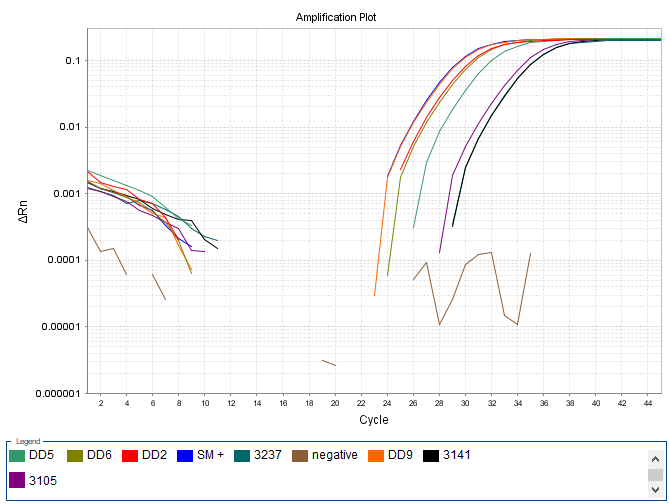 | 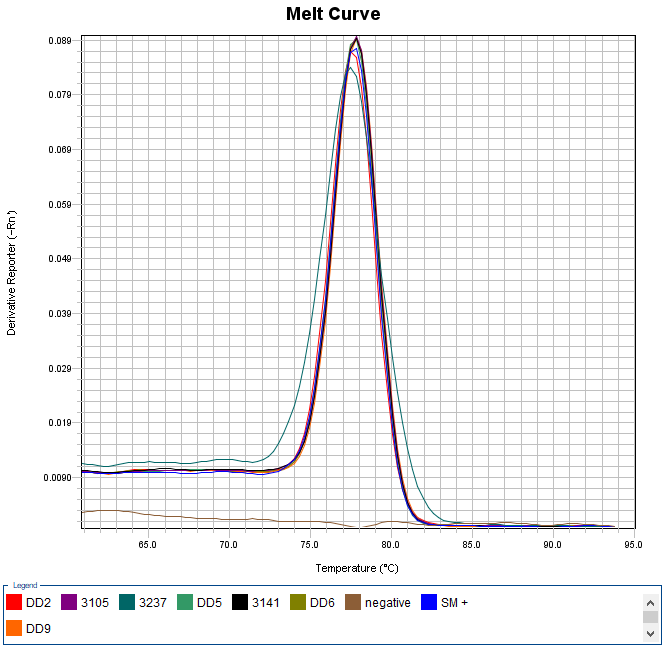 |

4) *brpA* gene

| Amplification plot | Dissociation curve |
| --- | --- |
| 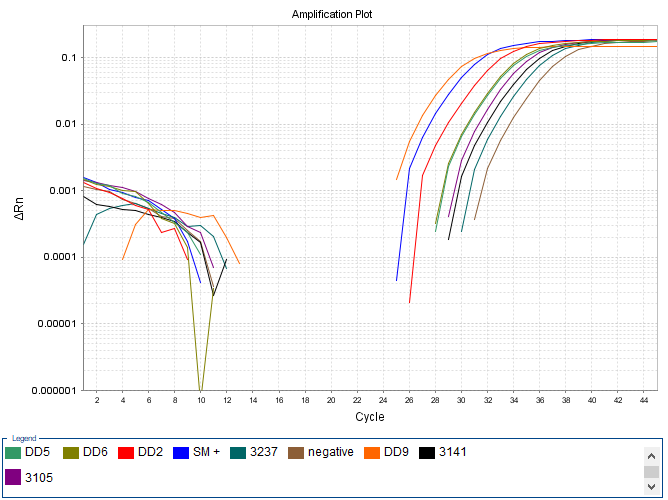 | 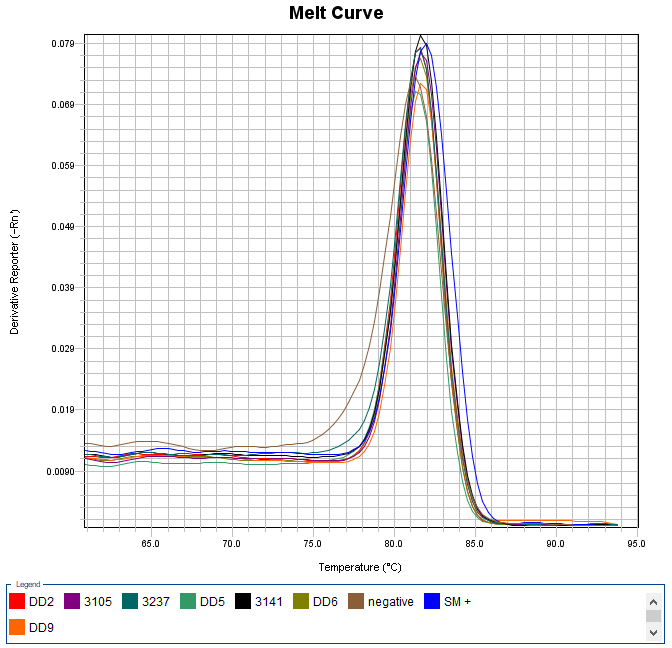 |

5) *comDE* gene

| Amplification plot | Dissociation curve |
| --- | --- |
| 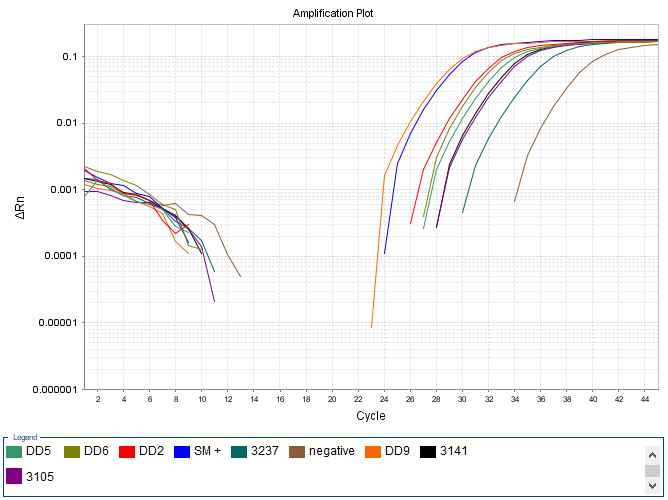 | 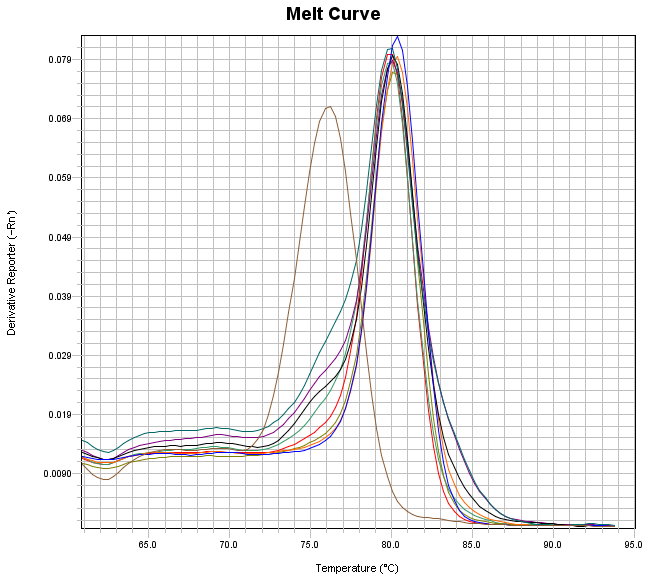 |

6) *vicR* gene

| Amplification plot | Dissociation curve |
| --- | --- |
| 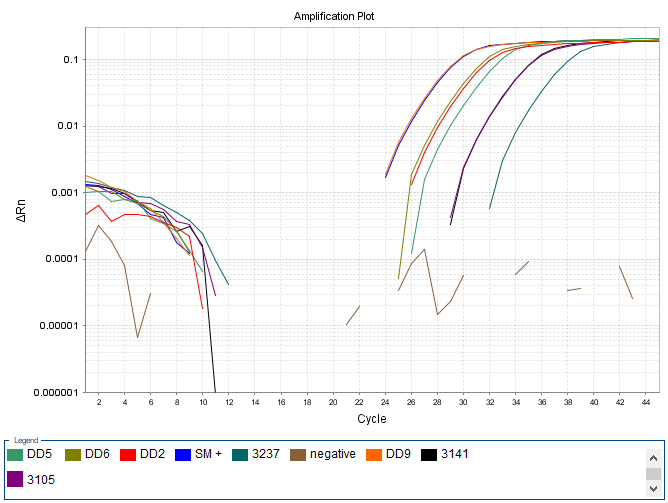 | 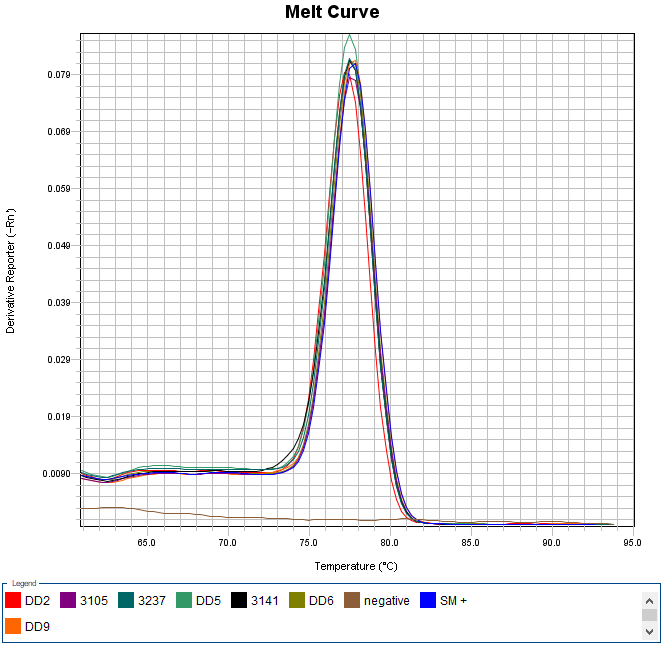 |

7) *gbpB* gene

| Amplification plot | Dissociation curve |
| --- | --- |
| 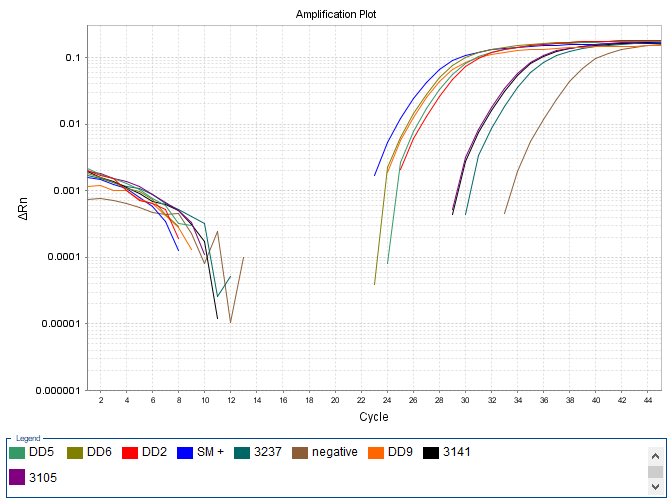 | 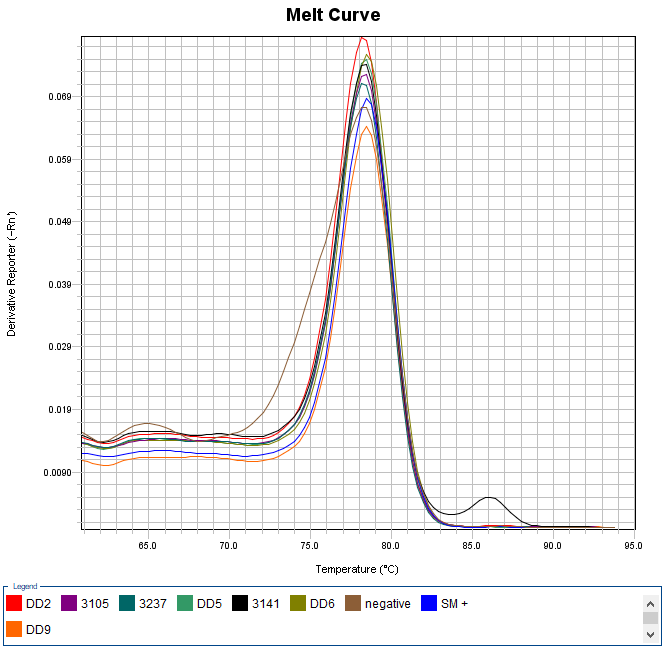 |

8) *spaP* gene

| Amplification plot | Dissociation curve |
| --- | --- |
| 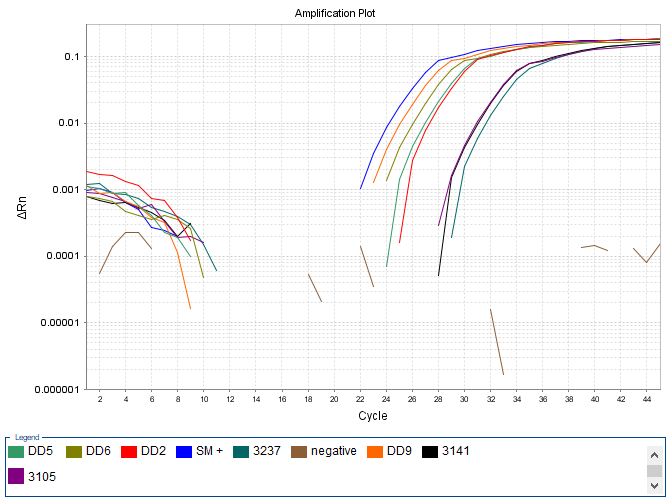 | 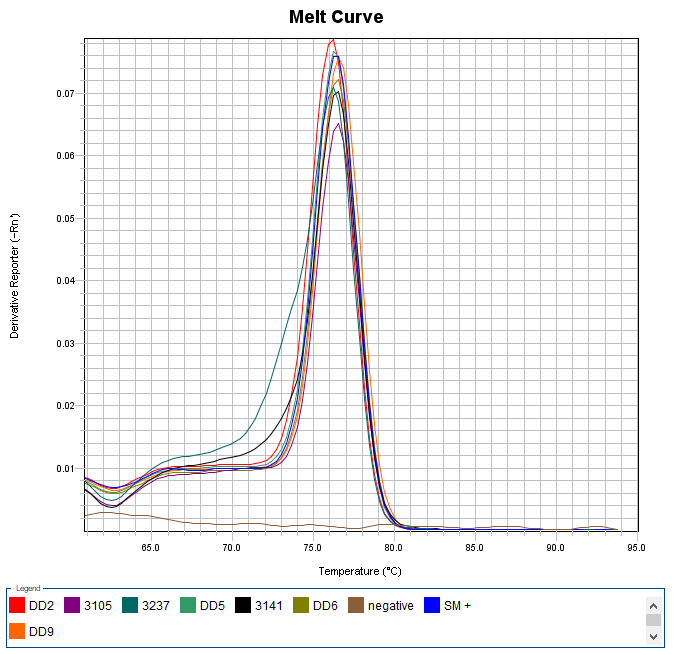 |

9) *16S rRNA* gene

| Amplification plot | Dissociation curve |
| --- | --- |
| 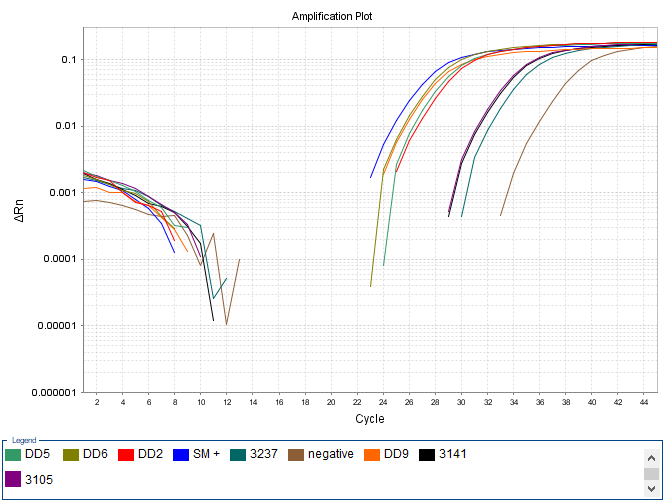 | 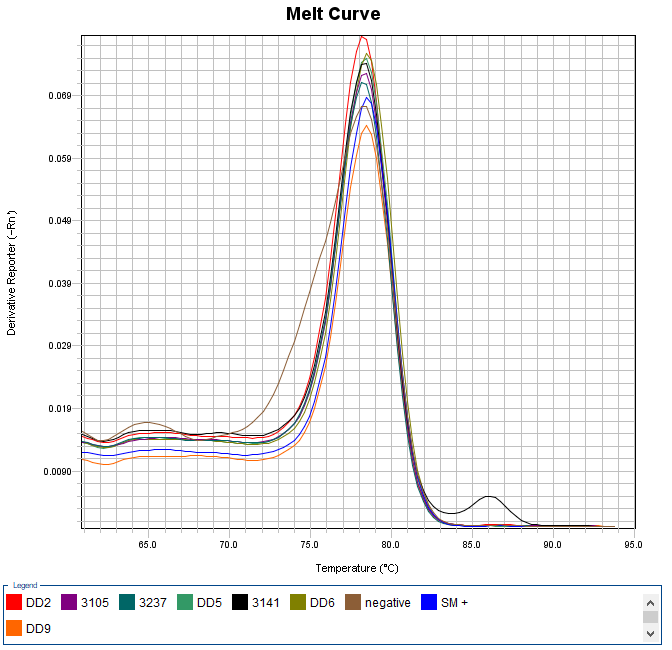 |
